# Influence of Actor's Congruent and Incongruent Gaze on Language Processing

**DOI:** 10.3389/fpsyg.2021.701742

**Published:** 2021-10-15

**Authors:** Dato Abashidze, Pia Knoeferle

**Affiliations:** ^1^Leibniz-Zentrum für Allgemeine Sprachwissenschaft (ZAS), Berlin, Germany; ^2^Department of German Studies and Linguistics, Humboldt-Universität zu Berlin, Berlin, Germany; ^3^Berlin School of Mind and Brain, Einstein Center for Neurosciences Berlin, Berlin, Germany

**Keywords:** tense comprehension, recent-event preference, incongruent gaze cue, eye-tracking, short-term linguistic and visual experiences

## Abstract

In interpreting spoken sentences in event contexts, comprehenders both integrate their current interpretation of language with the recent past (e.g., events they have witnessed) and develop expectations about future event possibilities. Tense cues can disambiguate this linking but temporary ambiguity in their interpretation may lead comprehenders to also rely on further, situation-specific cues (e.g., an actor's gaze as a cue to his future actions). How comprehenders reconcile these different cues in real time is an open issue that we must address to accommodate comprehension. It has been suggested that relating a referential expression (e.g., a verb) to a referent (e.g., a recent event) is preferred over relying on other cues that refer to the future and are not yet referentially grounded (“recent-event preference”). Two visual-world eye-tracking experiments compared this recent-event preference with effects of an actor's gaze and of tense/temporal adverbs as cues to a future action event. The results revealed that people overall preferred to focus on the recent (vs. future) event target in their interpretation, suggesting that while a congruent and incongruent actor gaze can jointly with futuric linguistic cues neutralize the recent-event preference late in the sentence, the latter still plays a key role in shaping participants' initial verb-based event interpretation. Additional post-experimental memory tests provided insight into the longevity of the gaze effects.

## 1. Introduction

Previous psycholinguistic research has shown that spoken language comprehension is highly sensitive to various linguistic and non-linguistic information sources. This adaptivity to both linguistic and visual information is evident in so-called “visual-world” studies in which participants' eye gaze is recorded in a visual context as they listen to related spoken utterances (e.g., Cooper, 1974; Tanenhaus et al., 1995; Chambers et al., 2002; Altmann and Mirković, 2009). During comprehension, language incrementally guides visual attention to objects and actions, and even to locations where an object or action was recently depicted (e.g., Spivey and Geng, 2001; Altmann, 2004; Knoeferle and Crocker, 2007).

For accounts that ground sentence processing in the immediate environment, one central issue has been how distinct linguistic and non-linguistic information sources guide interpretation and comprehenders' visual attention. Temporal linguistic cues, for instance, can point to recent or future events and verb meaning can identify what action is relevant for comprehension. However, different accounts make distinct predictions concerning the relative influence of verb-action reference and associations of temporal cues with events. The present paper reports the results of two visual-world eye-tracking experiments that assess to what extent grounded cues influence real-time language comprehension. One account of situated language comprehension (the Coordinated Interplay Account, Knoeferle and Crocker, 2007) predicts that verb-action reference should be preferred over relating non-referential linguistic (e.g., temporal) cues to scene events. Other accounts (Altmann and Mirković, 2009), by contrast, predict that no information source is given preference but instead that the current and earlier input influences what listeners anticipate (Altmann and Mirković, 2009, p. 589).

Among the language cues that rapidly guide attention is tense. For instance, listeners made more gaze shifts to the clipart depiction of an empty wine glass after they had been told that a woman *has drunk* wine than after they had been told that a woman *will drink* some wine (Altmann and Kamide, 2007). These looks emerged within very little time, at the onset of *the wine*. Tense also influenced listeners' visual attention in further research: Altmann and Kamide (2009) created an experimental setup in which listeners were either told that a *woman will put the glass onto the table* or that she is *… too lazy, to put the glass onto the table* (the glass was depicted as standing on the floor). Listeners then heard *… pour the wine carefully into the glass*. The situation conveyed by language influenced where listeners looked (more shifts of attention to the table when listeners had been told that the glass had been moved there than when they had been told it remained on the floor). These shifts in attention happened during *the wine carefully into*, before the glass was named. This suggests that the tense of the events conveyed by language rapidly influenced the unfolding interpretation, as evidenced by where people looked.

Further eye-tracking studies can speak to the interpretation of language about recent events compared with language about future event possibilities (e.g., Abashidze et al., 2011; Knoeferle et al., 2011). In these real-world eye-tracking experiments, language about a recent event was pitted against future-event reference. The prediction was that reference to a recent event should be preferred over anticipating plausible future events because of the absence of displaying future events in Experiment 1 of Abashidze et al. (2011) and Knoeferle et al. (2011). Unlike in prior research, tense had no immediate effect on spoken comprehension and visual attention: Listeners first saw an event acted out (e.g., an experimenter sugaring pancakes). Next, they heard either *Der Versuchsleiter zuckerte soeben die Pfannkuchen* (literally: “The experimenter sugared just now the pancakes”) or *.zuckert demnächst die Erdbeeren* (literally: “The experimenter sugars soon the strawberries”). Listeners ignored tense, and were more likely to look at the pancakes than at the strawberries during the verb and the ensuing adverb; they continued to do so as the strawberries were mentioned, and the preferential inspections continued even when future events were acted out equally often as the recent events (Abashidze et al., 2011; Knoeferle et al., 2011, Experiment 2). However, the equal number of future and recent events affected participants' looks toward the future-event target, as the looks to the future target started several hundred milliseconds earlier in Experiment 2 than in Experiment 1. It seems then that verb meaning trumped temporal cues in influencing how an utterance was related in real time to action events, providing a strong recent-event preference that is in line with evidence for the Coordinated Interplay Account (Knoeferle and Crocker, 2006, 2007).

These gaze patterns of the *overall* recent-event preference further replicated when future events were much more frequent within the experiment than recent events (Abashidze et al., 2019). In experiments by Abashidze et al. (2019), participants saw an actor sitting at a table with, for instance, cucumbers and tomatoes in front of him (other videos used different objects and sentences). First, the actor performed an action on one object (i.e., flavoring cucumbers) and then participants heard either *Der Versuchsleiter würzte kürzlich die Gurken*, literally: “The experimenter flavored recently the cucumbers” or they heard *Der Versuchsleiter würzt demnächst die Tomaten*, literally: “The experimenter flavors soon the tomatoes.” Afterwards, the actor flavored the tomatoes. The frequency with which participants were exposed to future events (and corresponding sentences in futuric present tense) relative to recent events was 75 vs. 25% and 88 vs. 12% in Experiments 1 and 2, respectively. Like in the earlier studies, participants ignored tense, and their eye-movements during sentence comprehension revealed a preference to inspect the target of the recent-event while and after hearing the verb. However, the frequency bias did modulate participants' visual attention (their attention to the future-event target started to increase 1,000 ms earlier compared with when recent and future events were acted out equally often, as in Experiment 2 by Knoeferle et al., 2011).

In summary, the within-experiment frequency of the futuric tense cues and action events seemed to matter less in guiding comprehension and visual attention than the strong bias of referential verb recent-event relations. Perhaps the attentional focus on the recent-action target was an eye-movement manifestation of the recency effect that has been documented in memory and cognition (Glanzer and Cunitz, 1966), as well as for visual attention (Zelinsky et al., 2011). Alternatively, listeners may have looked at the pancakes more because they were where the recent action was seen, and inspecting the location of the recent action helps ground the verb, a view that seems in line with grounding effects observed in the embodiment literature (e.g., Kaschak and Glenberg, 2000; Glenberg and Kaschak, 2002, also proposed by the Coordinated Interplay Account). Much research on grounding effects has focused on actions; but to more fully model comprehension we must also know how comprehenders reconcile recent actions with other cues that an actor can provide such as his/her eye gaze to objects and how incongruence in verb tense and actor gaze may affect processing. In fact, one cue that could swiftly guide a listener's visual attention to an object is the gaze shift of an interlocutor. Understanding which cues may be more dominant can permit us to develop mid-term a ranking of different linguistic information sources and their relation to the environment, generating further testable predictions.

### 1.1. Speaker/Actor Gaze Effects: Stimulus-Onset Asynchrony and Incongruence

Eye-tracking studies show that individuals are more likely to inspect a target object that a face had gazed at previously vs. an object that was not the recipient of a previous gaze (e.g., Mansfield et al., 2003; Frischen et al., 2007). For example, a study by Hanna and Brennan (2007) revealed that listeners followed a speaker's gaze shifts toward a target object before its mention (for the robustness of this finding across different settings see also e.g., Nappa et al., 2009; Knoeferle and Kreysa, 2012; Staudte et al., 2014; Sekicki and Staudte, 2018). Motivated by these studies, Experiment 3 by Abashidze et al. (2019) tested effects of an actor's gaze. If an actor's gaze matters more than verb-action reference, then listeners should as they hear the verb in the futuric tense, integrate tense and the actor's gaze and follow the actor's gaze to the future-event target. But if participants prefer to ground their inspection of objects in recent referentially-mediated events, they should overly prefer to inspect the target of the recently-seen event. The results revealed actor's gaze effects but these took some time to emerge (about 800 ms after gaze onset). We suggest that one reason for the arguably delayed effect could be that the actor shifted gaze only about 480 ms after the verb onset, which might not be an optimal temporal alignment with the other important cue, verb tense (as the onset of the tense cue that disambiguated between the simple past and the futuric present verb was at around 550 ms). The overlap between gaze and tense cues could hinder the immediate effect of the gaze. The timing of stimulus presentation can play a role in the timing with which these stimuli affect human language processing (e.g., for research on variation in stimulus onset asynchrony see de Groot et al., 1986; Rayner and Duffy, 1986; Friesen et al., 2005)^1^.

In addition to timing issues, the effects of gaze could be boosted or diminished depending on (in)congruence with language. Gaze incongruence with language has been examined (the actor either looked at a mentioned object or at another, language-mismatching object, Staudte and Crocker, 2011; Staudte et al., 2014). When the actor (in these studies, a virtual agent) inspected a mismatching object (a brown pyramid when a red one is mentioned), it was shown that the incongruency rapidly influences comprehension, as participants inspect target objects significantly less when they mismatch the gaze cue than when there is no gaze cue present. An eye-tracking study combining picture presentation with a sentence comprehension task, by Knoeferle and Crocker (2005), corroborates this mismatch effect for picture-sentence incongruence, indicated through faster reading times in congruent conditions (for related findings on gender stereotype effects in a picture-sentence task see Rodriguez et al., 2015)^2^. Experiments using picture-sentence verification tasks confirm the impact of incongruence, as matched picture-sentence pairs elicit faster responses than mismatched pairs (e.g., Carpenter and Just, 1975; Glenberg et al., 1999; Underwood et al., 2004). Overall, these findings show that both the timing of stimuli and the incongruence of an actor's gaze (but also of pictures) with language play an important role in language processing within the visual context.

### 1.2. The Present Experiments

As presented above, a speaker's eye-gaze direction can communicate information about what events or objects are mentioned next. The perception of an actor's gaze shift enables the listeners to direct their attention accordingly. But when speaker gaze mismatches language, it can also disrupt processing. Verb tense—much like speaker gaze—can activate anticipatory eye-movements toward a plausible target in a visual scene (e.g., Altmann and Kamide, 1999, 2007; Kamide et al., 2003). To add further details to accounts of situated language processing, we must arrive at a better understanding of how verb meaning, a recent event and an actor's gaze are reconciled and what listeners prioritize when these cues are in conflict. The present paper addresses these issues and reports the results of two visual-world eye-tracking experiments that contrast the effects of a recent action event with those of verb tense and actor gaze. In addition, we assessed the longevity of any such effects via short-term memory tests (see also Kreysa et al., 2018, for related research).

The causes underlying the preferential inspection of the recent event are unclear. Perhaps the preferential inspection is guided by the verb. The verb could be linked to representations of the recently inspected action and its location, prompting participants to shift gaze to the location of the action when they encounter the verb. A reduced recent-event inspection preference could emerge if the actor's gaze signals one target object (of a potential future action) but the (past) verb tense signals another target object (of the recently-seen action). Alternatively, a general recency effect (i.e., participants inspecting the target object of the recent action, independent of verb tense) could be observed. If this were the case, then a mismatch between the gaze shift and the verb tense should not interfere with the recent-event preference; however, gaze cues (e.g., the actor shifting gaze to the future target object during the expression of the verb) might diminish the recency effect by directing the listener's attention to the future target object.

The present experiments stress-tested listeners' preference for inspecting the target of a recent action by means of two changes. In Experiment 1, we reduced the stimulus onset asynchrony of actor gaze in relation to verb onset by having the actor shift gaze 400 ms earlier than in Experiment 3 of Abashidze et al. (2019). In Experiment 2, we created incongruence between the past tense verb and the actor's gaze by having the actor always look at the future-event target. Following the eye-tracking session, participants took part in a memory test. Most accounts of situated sentence comprehension focus on accommodating effects that unfold moment-by-moment during comprehension. The present research includes the consideration of the longevity of action and gaze effects with a view to ultimately bridging from accounts of comprehension to accounts of language learning. In Experiment 1, participants' task was to recall the sentence content on a per-constituent basis. In Experiment 2, participants' later memory of the visual information was tested. The aim of the memory tests is to provide more detailed insights into the recall of sentence content on a per-constituent basis and into the recall of the events. Previous studies reported a better recall of the future event (Abashidze et al., 2019, Experiment 3), which was not in agreement with the gaze data. However, the findings in the experiments that had increased the frequency of future over recent events revealed a better recall of the recent events (Abashidze et al., 2019), underscoring the recent-event preference in eye-movement data.

In Experiment 1, the gated memory test might reveal the higher recall of past (vs. future) tense sentences, if we replicate the recent-event preference. Alternatively, the gaze cue with its earlier onset compared with prior research (Abashidze et al., 2019) has a strong influence on visual attention and the anchoring of future events in working and short-term memory. If so, then we should see better recall for the future than recent-event target. If in Experiment 2, the incongruence (of the actor looking at the future-event target and the past tense verb referencing the different, recent-event target) affects the recent-event preference and if incongruence effects are long-lasting, then we might see a reduced recall performance for recent compared with future events in the memory test. Alternatively, the incongruence does not affect the recent-event preference and/or its effects are short-lived. If so, the recent (vs. future) events are anchored more firmly first in working and then in short-term memory and participants should be better at recalling the target of the recent (vs. future) events (Abashidze et al., 2019, Experiments 1 and 2 reported better recall of recent target objects).

## 2. Materials and Methods—Experiment 1

### 2.1. Participants

Thirty-two native German participants (11 males; age range: 19–31; *M* = 24.5 years, *SD* = 2.9 years, 1 male left-handed, 1 female left-handed), mostly students of Bielefeld University, took part in Experiment 1. Participants were paid 6 euros for their participation or received course credit. All had normal or corrected-to-normal vision, were unaware of the purpose of the experiment, and gave informed consent.

### 2.2. Materials and Design

Experiment 1 used the same experimental materials and design as Experiment 3 by Abashidze et al. (2019), but it modified the actor gaze cue onset time. While in the previous experiment, Experiment 3, the actor's gaze shift occurred on average 480 ms after the onset of the verb, in the current experiment, it occurred at verb onset. All experimental sentences had the structure NP-V-ADV-NP. In one condition, the sentence was in the past, with a verb in the simple past and a temporal adverb conveying the recent past. In the other condition, the sentence was in the futuric present tense (a present tense verb and a temporal adverb indicating the future). Note: An offline sentence completion study confirmed that the futuric present tense was as good a cue toward the future event as the alternative *will … VERB* future (see, Abashidze et al., 2019).

**Figure 1:**
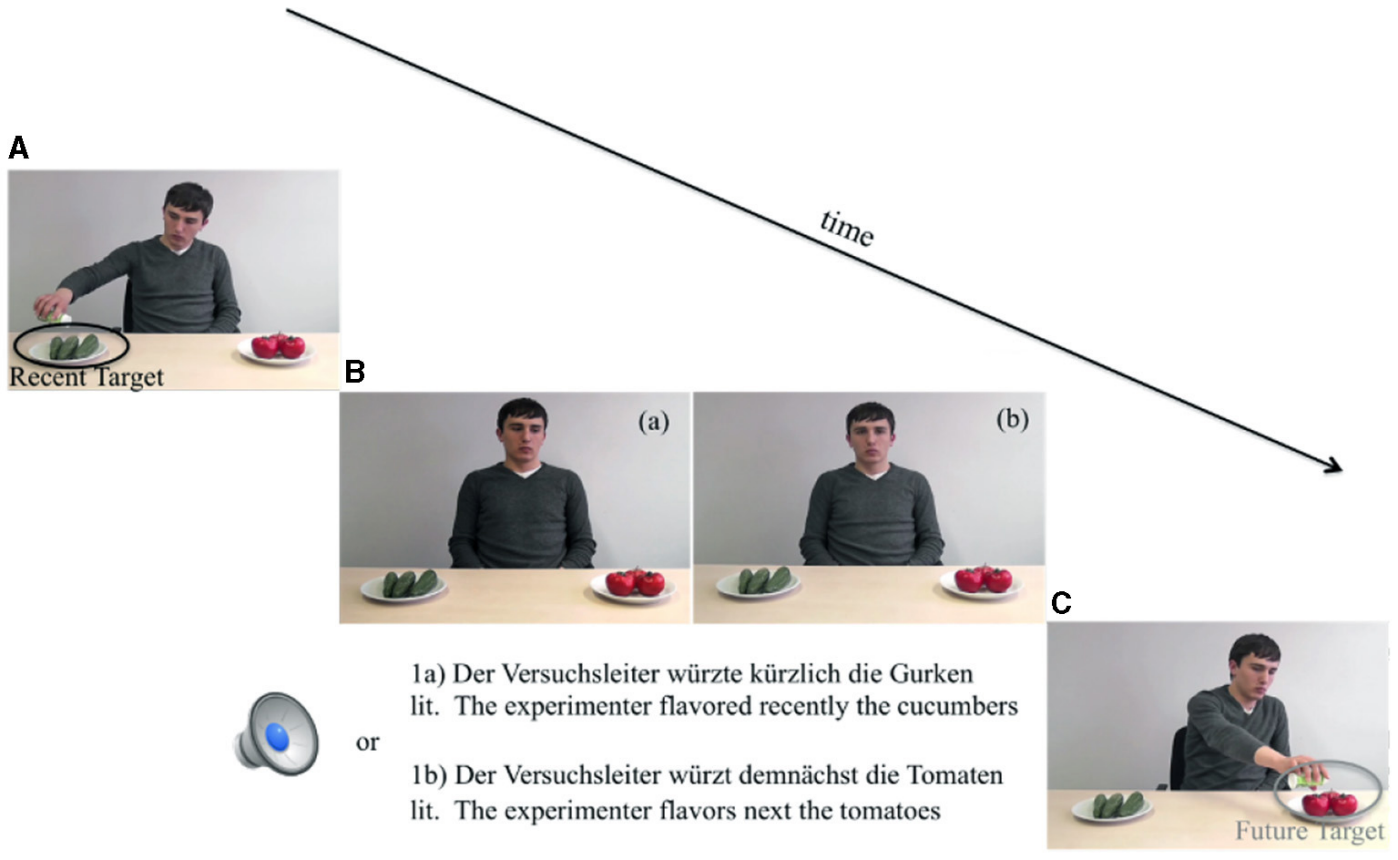
Sequence of events of a typical experimental trial. **(A)** Video of recent action for ca. 5 s. **(B)** (a) Static photo duration = 1,700 ms, gaze video and sentence duration + 700 ms; (b) Static photo + sentence duration + 700 ms. **(C)** Video of future action for ca. 5 s.

Figure 1 depicts snapshots from the videos for an example critical item (average duration of videos 5,015 ms). In Experiment 1, the first video showed the person carrying out an action on one object (e.g., flavoring cucumbers, Figure 1A)^3^. Then, in the no-gaze condition, a static photo, which was the last frame of the first video shown, the actor performed no action and looked straight ahead [Figure 1B(b)], and the sentence was played over the loudspeakers. Then, 700 ms after the sentence presentation ended, the second video showed the person carrying out the same action on another object (e.g., flavoring tomatoes, Figure 1C). In the gaze conditions, the actor's gazing video was played; from the verb onset, the actor gazed at the target object, which continued until the end of the sentence [Figure 1B(a)]. The actor's gaze to the future and past target object (when present) matched verb tense in the simple past and in the futuric present.

In addition to the experimental items (*N* = 24), we created 36 gaze videos for the filler items. Thus, each list had the 36 filler trials of which half featured a gaze video while the other half showed the static picture as the sentence was playing out. For one half of all trials (*N* = 30, 12 critical and 18 filler items), the experimenter gazed at the target object. In the other half of the trials, participants saw the experimenter in a static position (no gaze shift to the to-be-named future-target action occurred). The experimental and filler items were combined to form four lists using a Latin square design. Each list contained every critical item in only one condition and all of the fillers. Before the experiment, the lists were pseudo-randomized and each participant saw one version of the four lists.

### 2.3. Materials for Memory Test

For the gated memory test in Experiment 1, we created a three-stage procedure for each experimental sentence. The first stage only showed the first noun phrase and the verb stem. The second stage added the temporal adverb, and the third stage included three pictures. Two of these pictures were from the tested critical trial and the third was a distractor from another filler item.

### 2.4. Procedure

The procedure was similar to the procedure in Experiment 3 by Abashidze et al. (2019). An Eyelink 1,000 desktop head-stabilized tracker (SR Research) recorded participants' eye-movements. After participants read an information sheet and were verbally informed by the experimenter about the experiment and the methodology, their eyes were calibrated using a 9-point calibration grid. For each trial in the eye-tracking session, in Experiment 1, an action video was presented before the sentence (e.g., flavoring the cucumbers, see Figure 1A) and then a static picture [the last frame of the video, see Figure 1B(a)] appeared in the no-gaze condition, and after 700 ms, the sentence was played over the loudspeaker. On the contrary, in the gaze-condition, when the sentence started, at the verb onset, a gaze video began, in which the actor started to gaze either at the future target object (in the futuric present tense condition) or at the recent target object (in the past tense sentences condition) and the actor's gaze stayed on the target object until the sentence ended [see Figure 1B(a)]. The onset of the actor's gaze shift was set at the verb onset (note: verb onset was the average gaze detection time observed during a pre-test, see, Abashidze et al., 2019).

After the eye-tracking session, participants took part in the gated memory test in Experiment 1. They were assigned randomly to one of the four counterbalancing lists and each saw a different pseudo-randomized order of these lists. In Experiment 1, they were shown a Powerpoint slide with the first noun phrase and the verb stem, and they had to verbally complete the verb tense. The second stage on a next slide added the correct tense and the temporal adverb, participants were asked to recall the second noun phrase. If they were unable to do so, they received a further prompt at the third stage on another slide to select the correct referent out of three objects. After participants completed the memory test, they were then debriefed. The experiment lasted ~40–45 minutes.

### 2.5. Analysis

**Figure 2:**
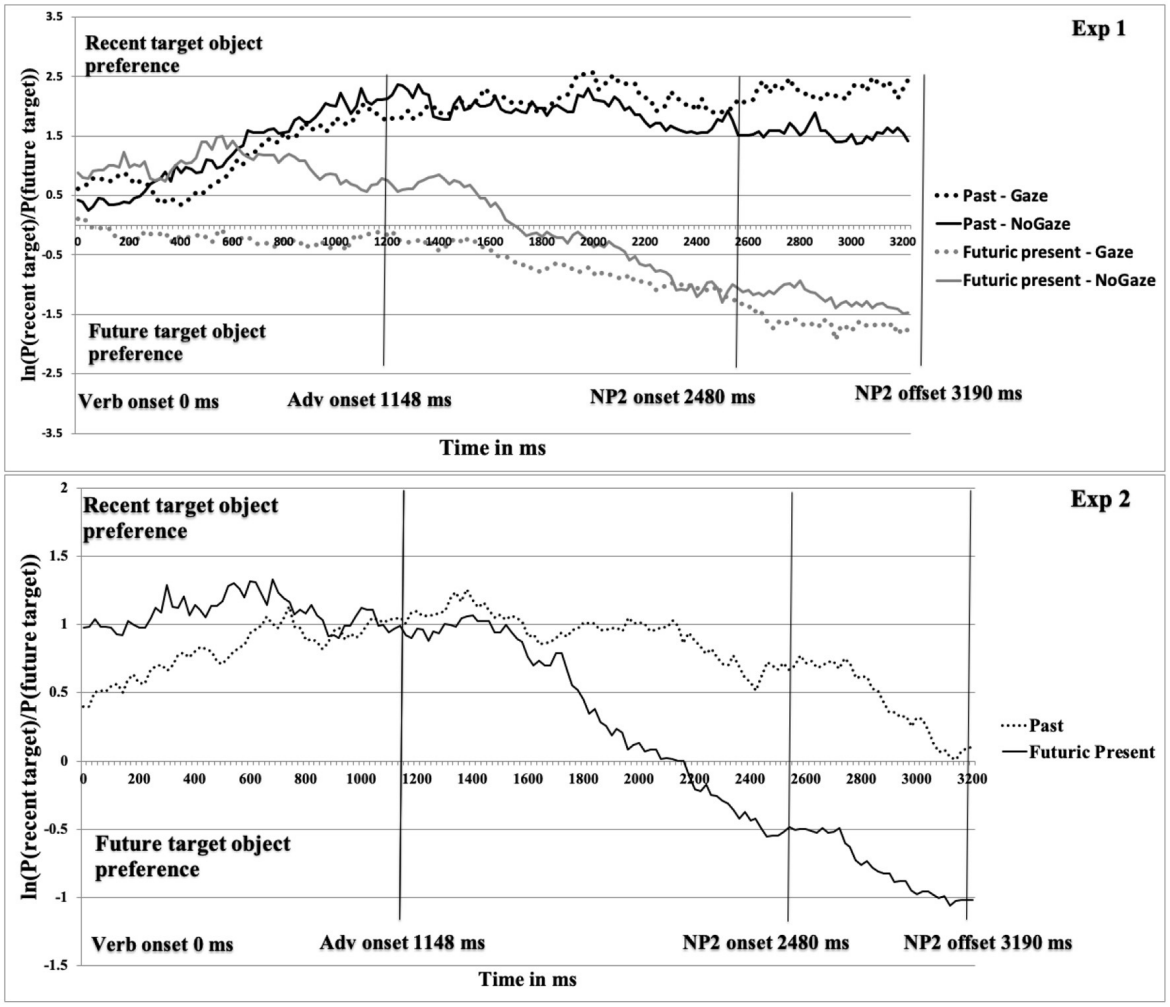
Mean log gaze probability ratios *ln* [*P* (recent target)/*P* (future target)] as a function of condition from Verb onset for Experiments 1 and 2. Reminding, Experiment 1 used Congruent Gaze in both sentence conditions. Experiment 2 used Incongruent Gaze in the past and No Gaze in the Futuric present condition.

For the *eye-tracking data*, we used the Data Viewer software (EyeLink Data Viewer, Version 1.11.900, SR Research) to prepare the eye-movement data for the analyses. We analyzed participants' fixations on two target objects, for which we created interest areas of the recent and future-event target (see Figure 1B). We set the critical time period between the onset of the verb and the end of the sentence. Next, we computed gaze probabilities to the two target objects in each successive 20 ms time slots. The amount of inspections to these interest areas are not linearly independent (more looks to one object mean fewer looks to the other, and vice-versa). We then computed mean log gaze probability ratios for the recent relative to the future target *ln* [*P* (recent target)/*P* (future target)]. For this calculation, an amount of fixations of zero implies that both targets are inspected equally often; a positive value means a preference for inspecting the recent target over the future target, and a negative ratio indicates the opposite (see, Arai et al., 2007; Knoeferle et al., 2011; Abashidze et al., 2019). By using this measure, we plotted the time course graphs from verb onset. As shown in Figure 2, Experiment 1, the light solid line indicates the future condition (sentence in the futuric present tense) and the dark solid line shows the recent condition (sentence in the past tense), while the light dotted line indicates the future gaze condition and the dark dotted line indicates the recent gaze condition.

The inferential analysis of the *eye-tracking data* for the current Experiment 1 with the two-way factorial design was identical to that for Experiment 3 in Abashidze et al. (2019). For this experiment, we defined the three time windows: the Verb region (from Verb onset until Adverb onset, mean duration = 1,148 ms); the Adverb region (from Adverb onset until NP2 onset, mean duration = 1,332 ms) and the NP2 region (from NP2 onset until NP2 offset, mean duration = 710 ms). The dependent variable was the mean log gaze probability ratios and the independent variables were tense and actor's gaze each with two levels, past vs. futuric present for tense, and gaze vs. no-gaze for actor's gaze in Experiment 1. In addition, to corroborate our results of the gaze cue onset distribution, we also performed a *post-hoc* between-experiment analysis comparing the gaze futuric present and gaze past tense condition of the present Experiment 1 with the gaze futuric present and gaze past tense condition of Experiment 3 from Abashidze et al. (2019).

For the *gated memory test*, we fitted a logistic linear mixed effect (LME) model to the binary (i.e., correct vs. incorrect) response data in Experiment 1. Since the most complex model failed to converge, we present analyses from models that exclude the three-way interaction of stage, tense, and gaze. Thus, in the model, the dependent variable was the response and the predictors were tense (past vs. futuric present) and actor's gaze (gaze vs. no-gaze). Subjects and items, with their intercepts and slopes, and the intercepts × slope interactions, were entered in the random effects of the model. Furthermore, the predictors were centered by transforming the fixed effect coding into a numerical value and centering it so that it had a mean of 0 and a range of 1 (Baayen, 2008). This effect coding has the advantage of allowing the coefficients of the regression to be interpreted as the main effects in a standard ANOVA (Barr, 2008).

### 2.6. Results

Descriptive results for the *eye tracking data* are presented in Figure 2, Experiment 1. In Experiment 1, the looks to the future and recent target object as a function of gaze started to diverge in the beginning of the Verb region in the gaze condition (~700 ms earlier than in Experiment 3 by Abashidze et al. (2019). In the no-gaze condition, the divergence began in the middle of Verb region. The early gaze cue in the Verb region triggered an increased number of looks to the future target object in the Verb region in both conditions. However, the recent-event preference was not reversed, as the log ratios remained at around zero in the gaze condition from the Verb onset until about 400 ms into the post-verbal region. By contrast, the looks in the futuric present no-gaze condition started to diverge in the middle of the Verb region and inspections remained above zero until the middle of Adverb region (until 1,700 ms after verb onset in Experiment 1). A clear increase in inspections of the future target object started at 1,600 ms in the gaze condition and at 2,100 ms in the no-gaze condition post verb onset.

The two-way ANOVA results indicated an effect of tense that was reliable in the analyses by-participants and by-items in all three regions *p*s < 0.000. Further, the analyses revealed actor gaze cue effects in the Verb region. However, in comparison to Experiment 3 by Abashidze et al. (2019), the gaze effect was eliminated in the Adverb region and similarly to Experiment 3 no effect was found in the NP2 region. Moreover, the conclusions from the descriptive analyses are mostly supported by the inferential analyses of the data. The log ratio indicated a positive value in all three regions (grand mean, between 0.37 and 1.15, see Table 1, Experiment 1) and the inferential analyses revealed a significant intercept in all three regions by-items and by-participants except the NP2 region *p*s < 0.068 (Table 2, Experiment 1). The tense effect in the Verb region revealed a significant difference in both by-participants *F*1(*d*f = 1,31) = 46.12, *p*1 = 0.000 and by-items analyses *F*2(*d*f =1,23) = 42.11, *p*2 = 0.000. Similarly, the effect of the gaze cues was significant in both by-participants *F*1(*d*f = 1,31) = 21.42, *p*1 = 0.000 and by-items analyses *F*2(*d*f = 1,23) = 5.67, *p*2 = 0.041. The significant effect of tense in the Verb region was replicated from Experiment 3 by Abashidze et al. (2019). The Adverb region revealed the tense effect in the by-participants *F*1(*d*f = 1,31) = 76.33, *p*1 = 0.000 and by-items analysis *F*2(*d*f = 1,23) = 93.82, *p*2 = 0.000. Similarly, the results for the NP2 region showed the tense effect in the by-participants *F*1(*d*f = 1,31) = 132.14, *p*1 = 0.000 and by-items analysis *F*2(*d*f =1,23) = 155.63, *p*2 = .000. Interestingly, the gaze cue effect emerged neither in the Adverb nor in the NP2 region. Moreover, the results in the present experiment did not reveal a reliable interaction of actor's gaze and tense by-participants in any of the three regions (in comparison to Experiment 3, by Abashidze et al., 2019). In the by-item analysis, the interaction was significant in the Verb region and marginal in the Adverb and NP2 regions. The marginal interaction indicates a more pronounced gaze cue effect in the future tense condition than in the past tense condition at least in the Verb and Adverb regions. Furthermore, *post-hoc* test for gaze compared with no gaze in the futuric present conditions showed a fully significant effect only in the Verb region *t*(31) = 3.72, *p* = 0.001, and a marginal effect in the Adverb region *t*(31) = 1.99, *p* = 0.056. The last region (NP2) did not show a reliable difference in inspections of the future target object (*p* = 0.734).

**Table 1:** Grand mean and mean log gaze probability ratios [ln (*P*(recent target)/*P* (future target)] by-participants as a function of condition and time region for the experiment.

**Regions**	**Future NoG**	**Future G**	**Past NoG**	**Past G**	**Grand mean**
**Experiment 1**					
Verb	1.25 (0.33)	−0.60 (0.34)	2.53 (0.35)	1.40 (0.36)	1.15 (0.20)
Adv	0.07 (0.36)	−0.73 (0.24)	3.46 (0.40)	3.32 (0.35)	1.53 (0.18)
NP2	−2.20 (0.42)	−2.37 (0.36)	2.56 (0.40)	3.47 (0.39)	0.37 (0.19)
**Experiment 2**	Futurir present		Past		
Verb	1.45 (0.30)		1.20 (0.28)		1.33 (0.21)
Adv	0.422 (0.19)		1.41 (0.29)		0.915 (0.17)
NP2	−0.793 (0.22)		.674 (0.26)		−0.059 (0.17)

*Standard errors (SE) in parentheses, Experiments 1 and 2*.

**Table 2:** ANOVA analyses for the eye-tracking data by regions: The intercept is also given since a significant intercept indicates that the grand mean is significantly different from 0, Experiments 1 and 2.

**Regions**	**Effect**	***F*1 (*df* = 1,31)**	***F*2 (*df* = 1,23)**	***P*** **1**	***P*** **2**
**Experiment 1**					
Verb	Intercept	36.06	42.30	0.000	0.000
	Tense	46.12	42.11	0.000	0.000
	Gaze	21.42	5.67	0.000	0.041
	Interaction	.754	12.70	0.392	0.002
Adv	Intercept	74.00	59.05	0.000	0.000
	Tense	76.33	93.82	0.000	0.000
	Gaze	2.59	0.47	0.118	0.500
	Interaction	1.53	3.61	0.226	0.070
NP2	Intercept	3.57	4.43	0.068	0.047
	Tense	132.14	155.63	0.000	0.000
	Gaze	0.931	1.913	0.342	0.180
	Interaction	2.65	4.09	0.113	0.055
**Experiment 2**					
Verb	Intercept	40.39	72.09	0.000	0.000
	Tense	0.37	0.59	0.544	0.448
Adv	Intercept	29.49	92.75	0.000	0.000
	Tense	7.29	15.55	0.011	0.001
NP2	Intercept	0.12	0.11	0.734	0.743
	Tense	18.62	24.54	0.000	0.000

In order to examine the effect of the early actor gaze shift further, we compared the current Experiment 1 and Experiment 3 of Abashidze et al. (2019). As described above, in the current experiment, participants in the gaze futuric present condition started to inspect the future-event target around 200 ms after gaze onset. By contrast, in Experiment 3 of Abashidze et al. (2019), participants shifted attention toward the future-event target around 500 ms after the actor gaze onset. We conducted an independent-samples *t*-test on the mean log-gaze ratios of the combined word regions (Verb, Adv and NP2) in the futuric present sentence condition, separately with and without gaze cue. We also ran an independent-samples *t*-test on the mean log-gaze ratios of the gaze and no-gaze condition in the Verb region in the futuric present sentence condition. The number of participants and critical items were equal in both experiments. The first *t*-tests were not significant in the gaze condition and marginal in the no-gaze conditions *t*(62) = 1.77, *p* = 0.081, respectively. Similarly, the second set of *t*-tests showed a marginal effect *t*(62) = 1.73, *p* = 0.087. These findings corroborate the results of the other comparisons, demonstrating that the timing of gaze onset manipulation in the current Experiment 1 (vs. Experiment 3 of Abashidze et al., 2019) marginally reduced the preferential inspection of the recent-event target in the futuric present tense condition.

While previous studies by Abashidze et al. (2019) showed a significant intercept in the Verb, Adverb, and NP2 regions, the current study did not reveal a fully significant difference in the NP2 region (except the marginal effect *p*s < 0.068 by participants analyses, see Table 2), suggesting that the early gaze cues toward the future target object eliminated the overall significant recent-event preference in the NP2 region. Importantly, the conclusions emerging from the descriptive analyses above are in line with the inferential analyses of the data. Thus, the current manipulation had an earlier and stronger effect but it could not override the overall recent-event preference in early word regions. In contrast to Experiment 3 of Abashidze et al. (2019), no clearly reliable interaction of gaze and tense emerged in the current experiment (the gaze cue effect did not differ significantly in the past and future tense condition).

**Figure 3:**
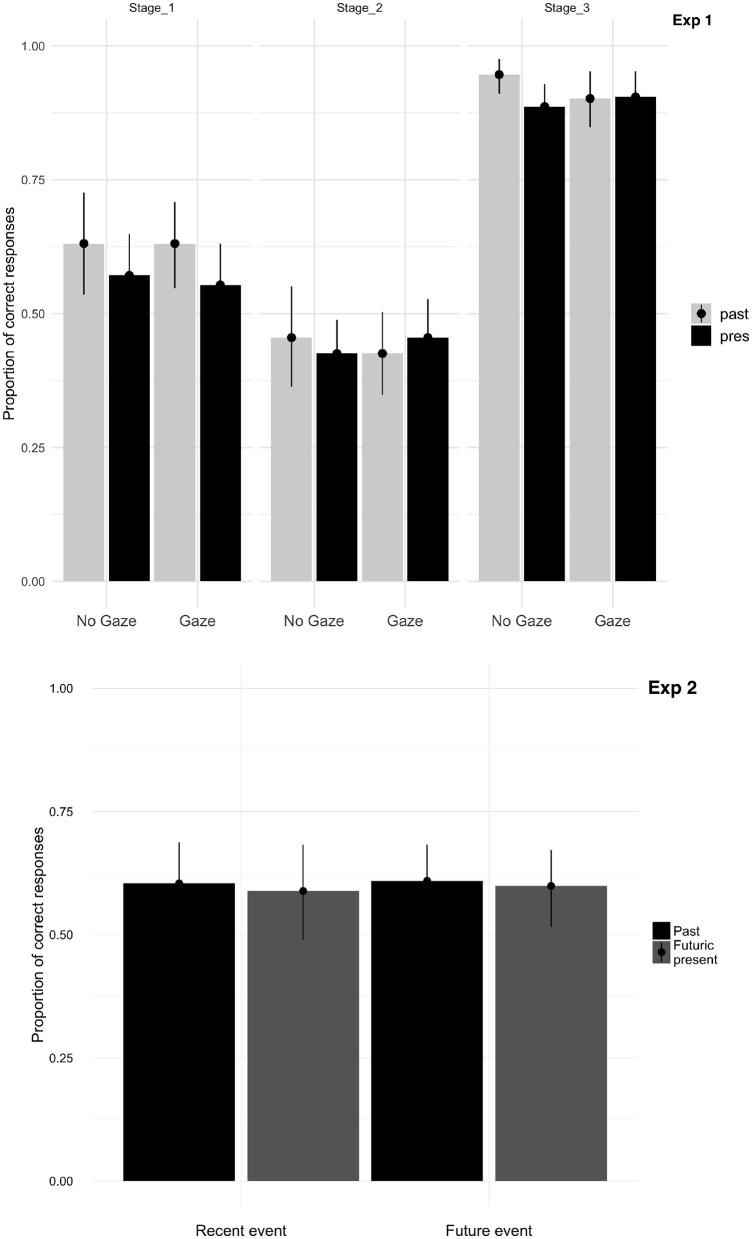
Proportion mean of correct answers as a function of stages, gaze, and tense in Experiment 1 and as a function of object and tense in Experiment 2 (with error bars plotting the standard error of the mean).

The results of the *gated memory test* (average percentages by participant) are displayed in Figure 3, Experiment 1. Participants overall correctly answered 64% of the questions from all three stages. They were more accurate at stage one (59%) than two (43%), and accuracy was highest at stage three (with 90%). The LME analyses for stage three showed an effect of tense (*p* < 0.003, higher accuracy for past than future tense conditions) and of gaze cue (*p* < 0.01, higher accuracy without than with gaze), in the absence of an interaction.

## 3. Materials and Methods—Experiment 2

### 3.1. Participants

Another thirty-two native German participants (8 males; mean age = 24.2 years, *SD* = 2.8 years, age range: 19–29; 2 male left-handed, 1 female left-handed), from Humboldt University of Berlin, took part in Experiment 2. Participants were paid six euros for their participation or received course credit. All had normal or corrected-to-normal vision, were unaware of the purpose of the experiment, and gave informed consent.

### 3.2. Materials and Design

Experiment 2 used the same experimental materials and design as Experiment 1, but it reversed actor's gaze cue in the past tense condition and no gaze cue was shown in the futuric present condition. Figure 1 depicts snapshots from the videos for an example critical item with a mismatching condition in Figure 1A(a). Further, the 36 filler trials from the first experiment were used in the second experiment, in which, in order to balance the number of the incongruent gaze videos in the past and futuric present tenses, 12 filler items featured a mismatching gaze video in the futuric present tense condition. The experimental and filler items were combined to form four lists using a Latin square design. Each list contained every critical item in only one condition and all of the fillers. Before the experiment, the lists were pseudo-randomized and each participant saw one version of the four lists.

### 3.3. Materials for Memory Test

In Experiment 2, we created two snapshots of the first and second video of each experimental item, i.e., showing the experimenter performing one of the two actions (see Figures 1A,B). The two snapshots associated with each item were combined into one display and shown to participants. Two versions were created in which the respective location of the two pictures was counterbalanced. While in the first experiment, the gated memory test was introduced to gain more insight into the later recall of the linguistic components on the one side, in the second experiment, the memory test aimed to observe the recall of the visual information on another side and their possible correlation with the eye-movement data.

### 3.4. Procedure

The procedure was similar to the procedure in Experiment 1. Participants viewed the first video showing the person carrying out an action on one object (e.g., flavoring cucumbers, Figure 1A)^4^. In the past tense condition, an incongruent actor's gaze video was played from the verb onset, which continued until the end of the sentence [Figure 1B(a)]—actor gazed at the future target -tomatoes- so the gaze mismatched the past tense verb. By contrast, in the futuric present tense condition, a static photo (the last frame of the video) was shown. In that frame, the actor performed no action and looked straight ahead [Figure 1B(b)], and the sentence was played over the loudspeakers. Then, 700 ms after the sentence presentation ended, the second video showed the person carrying out the same action on another object (e.g., flavoring tomatoes, Figure 1C).

After the eye-tracking session, participants took part in the memory test. In Experiment 2, participants were exposed to pictures such as those in Figures 1A,B, one for each experimental item. Above the picture, a question was shown in one of two versions: (a) *Welche Aktion wurde VOR dem Satz durchgeführt?* “Which action was performed before the sentence?” or (b) *Welche Aktion wurde NACH dem Satz durchgeführt?* “Which action was performed after the sentence?” Participants answered the questions by a button press. They pressed the left-hand button on a button box if they thought the left picture was correct and the right button if they thought the right picture was correct. The left/right button press for the correct answer was counterbalanced for the items in each list. After participants completed the memory test, they were then debriefed. The experiment lasted ~40–45 minutes.

### 3.5. Analysis

For the *eye-tracking data*, we used the same method to analyze the eye-movements as in Experiment 1. We computed mean log gaze probability ratios for the recent relative to the future target *ln* [*P* (recent target)/*P* (future target)]. Then, we plotted the time course graphs from Verb onset, as shown in Figure 2, Experiment 2. The solid line indicates the future condition and the dotted line shows the recent condition. An amount of fixations of zero indicates that both targets are inspected equally often; a positive value means a preference for inspecting the recent target over the future target, and a negative ratio indicates the opposite. The inferential analysis of the eye-tracking data for Experiment 2 with the one-way factorial design was identical to that for Experiments 1. The dependent variable was the mean log gaze probability ratios and the independent variable was the tense with two levels, past vs. futuric present. In addition, we performed a *post-hoc* analysis by comparing the results of congruent gaze cues of Experiment 1 with the results of incongruent gaze cues of Experiment 2 in the past tense conditions.

For the *memory test data*, we fitted a logistic linear mixed effect (LME) model to the binary (i.e., correct vs. incorrect) response data of the memory test. In this model, the dependent variable was the response and the predictors were tense (past vs. futuric present) and target event (recent vs. future target). Further, subjects and items, with intercept and x interaction, were conducted in the random effects of the model.

### 3.6. Results

Descriptive results for the eye tracking data are presented in Figure 2, Experiment 2. In Experiment 2, the looks begin to diverge before the mid-Adverb region in the past and futuric present tense condition (note: when the actor gaze mismatched, people spent less time looking toward the recent target in the past than in the future condition). In the future condition, participants then rapidly decreased the inspection of the recent-event target, and within 500 ms they preferentially inspected the future-event target. The larger increase in looks toward the future target, which continued in the following NP2 region, reached a negative grand mean in the last region. In other words, in the NP2 region, participants overall inspected the future target object more than the recent target object (see, Figure 2, Experiment 2).

The one-way ANOVA results did not reveal an effect of tense in the Verb region neither by-participants *F*1(*d*f =1,31) = 0.37, *p*1 = 0.544 nor by-items *F*2(*d*f = 1,23) = 0.59, *p*2 = 0.448. However, the Adverb region showed a clear significance in both by-participants *F*1(*d*f = 1,31) = 7.29, *p*1 = 0.011 and by-items analysis *F*2(*d*f = 1,23) = 15.55, *p*2 = 0.001. Similarly, the NP2 region revealed a fully significant tense effect in both by-participants *F*1(*d*f = 1,31) = 18.62, *p*1 = 0.000 and by-items analysis *F*2(*d*f = 1,23) = 24.54, *p*2 = 0.000. The pattern of inspections to the future target object in these regions is similar to that in previous studies (see for instance Experiment 1 by Abashidze et al., 2019). Moreover, the disappearance of the significant intercept in the NP2 region suggests that the incongruent gaze cue in the past tense condition did indeed affect the recent-event preference (by removing its significant effect, which was present in all previous experiments i.e., by Knoeferle et al., 2011; Abashidze, 2017; Abashidze et al., 2019).

Further analyses determined the strength of the recent-event preference in the gaze and no-gaze futuric present tense condition in Experiment 1 and in the futuric present tensed condition in Experiment 2. One-sample two-tailed *t*-tests evaluated whether the mean log ratios of participants and items were significantly different from zero (Table 3, Experiments 1 and 2). In Experiment 1, the log ratio means in the gaze condition in all word regions are negative between −1.76 and −6.61 (showing a preference for the future target). The *t*-tests also reached full significance in all three regions *p* = 0.005, except a marginal effect by-participants analyses in the Verb region *p* = 0.089. In the no-gaze condition, the log ratio means are positive in the Verb and Adverb regions between 0.204 and 5.09 (showing a preference for the recent target), but the means turn into the negative −5.02 in the NP2 region. The *t*-tests revealed a significant difference of the mean log ratios from zero in the NP2 region *p* = 0.001 (preference for the future target) and no significant difference was found in the Adverb region. But that difference was significant in the Verb region *p* = 0.001 (a preference for the recent target). Similarly to the no-gaze condition, in Experiment 2, the log ratio means are positive between 2.19 and 6.61 in the Verb and Adverb regions (showing a preference for the recent target), which were confirmed by the significant *t*-test *p* = 0.005. But the log ratio means turn into the negative in the NP2 region and *t*-tests turned significant *p* = 0.001 (corroborating a preference for the future target). Moreover, the comparison between Experiments 1 and 2, by the independent *t*-tests revealed no reliable effect in the Verb region, but the effects were significant in the Adverb and NP2 regions *ps* = 0.000. This suggests that participants inspected the recent-event target significantly less in the incongruent than congruent gaze condition.

**Table 3:** One-sample two-tailed *t*-tests on the log ratio means for the futuric present gaze and no-gaze conditions, testing whether these are significantly different from 0 by regions, in Experiments 1 and 2.

**Regions**	**Gaze**	***t*1 (*df* = 1,31)**	***t*2 (*df* = 1,23)**	***P*** **1**	***P*** **2**
**Experiment 1**					
Verb	Gaze	−1.76	−2.43	0.089	0.023
	No gaze	3.81	5.09	0.001	0.000
Adv	Gaze	−3.07	−3.07	0.004	0.005
	No gaze	.204	0.401	0.840	0.692
NP2	Gaze	−6.61	−5.63	0.000	0.000
	No gaze	−5.21	−5.02	0.000	0.000
**Experiment 2**					
Verb		4.79	6.61	0.000	0.000
Adv		2.19	5.81	0.036	0.000
NP2		−3.52	−3.90	0.001	0.001

The findings of the memory test in Experiment 2 are presented in Figure 3, Experiment 2. Participants correctly answered questions on average with 61%. They were slightly more accurate in recognizing the future (matching) events (60%) than the recent (mismatching) events (59%). The LME analyses did not reveal any significant difference in recalling the recent vs. future events.

## 4. Discussion

Two visual-world eye-tracking experiments and post-experiment memory tests assessed language users' tendency for the recent-event preference (e.g., Knoeferle et al., 2011; Abashidze, 2017; Abashidze et al., 2019) by pitting it against immediate matching and mismatching gaze cues. We investigated: (a) whether the recent-event preference is affected by the timing of the gaze cue onset when the actor's gaze shift started early at the verb onset and remained throughout the utterance, (b) whether a verb-tense mismatching gaze shift can reduce or even eliminate the preferential inspection of a recently acted-upon target object. Our prediction was that if the recent-event preference is a strong contextual behavior, which might be partly guided by the verb, then participants' overall preference for inspecting the recent-event target should disappear as follows: First, it should disappear when participants realize that the actor's gaze cue accompanying the futuric present tense from the verb onset goes toward the future target in Experiment 1. Second, it should disappear, when participants notice the mismatch between the past tense verb referring to the recent action and the actor's gaze shift (toward a future-action target) in Experiment 2. Eye-tracking results revealed that participants overall preferentially inspected the recent-event target in replication of recent-event preference patterns (e.g., Knoeferle et al., 2011; Abashidze et al., 2019), except in the last word region, in which the significant intercept disappeared. The early gaze cue clearly affected participants' visual activity in the future tense condition, and they made anticipatory eye-movements toward the future target more early and often than otherwise. These results support accounts that prioritize the grounding of verb reference in a recent action over relating non-referential tense cues to another plausible action event; as a result, we must assume a ranking of information sources and not just that the current and earlier input influences what listeners anticipate. Some support for this view also comes from findings by Altmann and Kamide (2007) in their experiment on tense effects. The results showed, as discussed, a modulation of listeners' visual attention by tense cues in the sentence (more looks to a table when a glass was described as having been moved there than when it was described as not having been moved). But at the same time, the actual glass received more looks than the destination of the moving action, even when the description indicated the glass had been moved. This could be viewed as an instance of prioritizing that which has been immediately depicted over a mental representation of future-event possibilities, much in line with the recent-event preference. Turning back to our results, the recent-event preference is not absolute; when it clashed with an actor's gaze and temporal linguistic cues pointed toward a future event, it was neutralized. This suggests that when two cues (e.g., the actor's gaze and temporal cues) which conflict the recent-event cue are present, the recent-event preference is eliminated, a prediction that can be tested for other cues and world-language relations in future research.

In Experiment 1, the gaze shift coinciding with verb onset had an earlier effect (compared to Experiment 3 by Abashidze et al., 2019, when the gaze shift occurred 480 ms after verb onset) particularly in the future tense condition. In that condition, participants had already started to inspect the future target, 160 ms after the gaze cue onset (Figure 2, Experiment 1). In fact, looks to the recent and future targets were evenly distributed (slightly below the chance level). That pattern persisted throughout the verb until the middle of the adverb. Only after ~1,600 ms from the verb onset, did looks toward the future target increase continuously. By contrast, in the no-gaze future condition, participants preferentially looked toward the recent-event target until the middle of the adverb region of the sentence. These findings of an immediate actor's gaze effect in the futuric present tense condition is in line with other findings (e.g., Driver et al., 1999; Donk and van Zoest, 2008; Wang and Apperly, 2017) that reported a short-lived effect of such situation-specific cues in language processing.

The timing of the gaze shift in relation to the verb onset mainly influenced the future tense condition. In the past tense conditions, actor gaze effects on the listeners' attention were less pronounced and less immediate than in the future condition. Perhaps, inspection of the recent target during the verb in Experiment 1 (when the acted-upon recent event and the past tense verb that referred to it created a clear bias toward the recent target) was already so robust that the additional gaze cue did not lead to a further increase in looks toward the recent target (see also Abashidze et al., 2019, Experiment 3). Unlike in the future condition, it was only at the end of the adverb that gaze triggered more looks to the recent target in the gaze condition than in the no-gaze condition in Experiment 1 (black dotted and solid lines in Figure 2), with this increase lasting until the end of the sentence. Since gaze strongly evidences the upcoming mention of objects, a more immediate and full reversal of the recent-event preference might have been expected (at least in the future tense condition). However, even the very early effects of gaze in the future condition of Experiment 1 (Figure 2, Experiment 1) did not lead to a sudden reversal of the fixation preference; in fact, during the initial 1,450 ms, the log ratio hovered around zero, suggesting strong competition for preference. Furthermore, the gaze conditions did not differ significantly whether the actor gaze shift had its onset 480 ms after the verb or at verb onset, as was the case in the present experiments (comparing the present Experiment 1 and Experiment 3 of Abashidze et al. (2019).

In Experiment 2, eye-movement results did not indicate an early preferential inspection (for instance, in the verb and adverb regions) of the future-event target, despite the incongruence between the past tense verb and actor's gaze. Nevertheless, at the onset of the incongruent gaze during the verb, people made less inspections to the recent target in the (mismatching) past than in the futuric present condition. Past research has shown a strong influence of incongruence in language comprehension (e.g., Sturt, 2003; Kelly et al., 2010; Staudte and Crocker, 2011). In line with past results, we expected that the incongruence could make participants believe that the gazed-at future target object would be mentioned next, and that this expectation could reduce participants' looks toward the recent target at least until its name was heard. But this is not what we observed and we instead replicated the recent-event preference. The inspection of the future target in the futuric present condition started only at 2,200 ms after the verb onset. These results show a similar preferential pattern as our previous findings when we manipulated frequency (for instance, Abashidze et al., 2019, in Experiment 2).

Previous studies detected an immediate speaker's gaze effect robustly across different settings (e.g., Hanna and Brennan, 2007; Nappa et al., 2009; Kreysa and Knoeferle, 2013; Sekicki and Staudte, 2018). By comparison, in the current Experiment 2, in the past tense condition (with the presence of mismatching gaze), participants ceased with the inspection of the recent-event target only at the end of the sentence (i.e., after mention of the future target). Thus, they ignored that the actor's gaze had been directed at the future target from the verb onset. This indicates a strong reliance on the recently-seen event and a relatively slow effect and weak influence of the incongruent actor's gaze when competing with the preceding recent-event action referenced by the verb. However, the *post-hoc* comparison for matching and mismatching gaze in the past tense condition between Experiment 1 and Experiment 2 revealed reliable effects in the Adverb and NP2 regions, as participants inspected the recent-event target significantly less in the incongruent condition (in Experiment 2) compared with the congruent condition (in Experiment 1).

Previous research has applied a human gaze cue in different paradigms, for instance, in reflexively cueing human attention (e.g., Mansfield et al., 2003; Bayliss and Tipper, 2006; Frischen et al., 2007; Böckler et al., 2011) and to influence sentence comprehension (e.g., Hanna and Brennan, 2007; Nappa et al., 2009; Knoeferle and Kreysa, 2012; Staudte et al., 2014; Sekicki and Staudte, 2018). A person's gaze to an object can matter at the perceptual level but it also has emotional implications for viewers. Data from a gaze-cuing task (Bayliss and Tipper, 2006) revealed that participants were far more likely to interpret a face as reliable or trustworthy when it looked repeatedly toward an upcoming object than when it looked away from a target object. When an emotional expression was added to the gaze-cueing experiment, happy faces increased the effect of trustworthiness, whereas angry faces were seen as having been viewed more often (Bayliss et al., 2009). Neutral faces produced effects of trustworthiness that fell somewhere in between those of the positive and negative faces. Interestingly, viewers consistently followed gaze direction, irrespective of the emotional valence and perceived trustworthiness of the facial stimuli. This evidence corroborates the view that another person's gaze is a strong cue in cueing tasks and during language processing (see Macdonald and Tatler, 2013, for a social interaction task). Here, participants were robustly influenced by the presence of a person's gaze cue vs. its absence. A reaction time experiment by Böckler et al. (2011) showed that shared attention of human faces (two faces looking at each other) exerted an acceleration effect on participants' ability to correctly select a target object. However, this effect was not produced when the shared attention of the facial stimuli was directed away from the upcoming object. These insights into the robust effects of a person's object-directed gaze on the behavior of onlookers suggest that gaze is an influential cue in language and cognition. The finding that the recent-event preference replicated even when the actor's gaze mismatched suggests that the preference is truly robust, and corroborates the view that different information sources are not all equal but ranked in importance even if that ranking is relative and not absolute.

The post-experimental gated memory test following Experiment 1 yielded mixed findings: On the one hand, the findings were in line with recent-event preference patterns (Figure 3, Experiment 1), as participants recalled past tense sentences better than futuric present tense sentences in the first and last stage; but recall was not reliably better for the past sentences in the second stage, countering the recent-event inspection preference (see also Abashidze and Knoeferle, 2015). On the other hand, while gaze (vs. no-gaze) was beneficial to enhance attention toward future targets in the future condition, it did not enhance futuric present tense sentence recall (Figure 3, Experiment 1). A possible explanation for this is that gaze is only used ‘on the fly’ with short-lived effects on cognitive processing (see also, Wang and Apperly, 2017; Kreysa et al., 2018). The better recall of stimuli in the past condition in Experiment 1 could be explained by the congruent recent-events and language, evoking more in-depth processing and increased attention to the stimuli, thus benefitting the later recall of event information. In Experiment 2, no reliable difference in recall emerged for recent vs. future events; this could be because of possible short-lived effects of the mismatching gaze.

The present findings clearly showed that early gaze cues modulated the recent-event preference earlier in the sentence than short-term frequency biases toward future events. But speaking to the robustness of the preference, even an immediate shift in the actor's gaze at the verb did not immediately reverse this preference. These results corroborate that the recent-event inspection preference is robust. Moreover, the recall accuracy in the post-experimental memory test in Experiment 1, suggests that the matching gaze cue did not influence participants' short-term memory of the recall of the sentence tense (at least not in all stages). Gaze mismatches, by contrast, seem to have had effects in the sense that they influenced tense effects. But the overall reduced inspection preference, which mainly occurred in the last region (NP2), did not translate into recall of the stimuli. The conflicting memory-test results suggest the need for further research assessing the functional contribution of this attentional preference to later recall of events from recent memory.

## Data Availability Statement

The raw data supporting the conclusions of this article will be made available by the corresponding author (DA), without undue reservation.

## Ethics Statement

For Experiment 1, ethical review and approval was not required for the study on human participants in accordance with the local legislation and institutional requirements effective at the time the experiment was conducted. The patients/participants provided their written informed consent to participate in this study. Written informed consent was obtained from the individual(s) for the publication of any potentially identifiable images or data included in this article. Experiment 2 (conducted November 2016 - January 2017) was covered by the laboratory ethics vote of the psycholinguistics group at the Humboldt-Universität zu Berlin (valid 17.09.2016-16.09.2022).

## Author Contributions

DA and PK designed the experiments and revised and edited the manuscript for its complete form. DA implemented, conducted, and analyzed the experiments and prepared a first draft. All authors contributed to the article and approved the submitted version.

## Funding

This research was funded by the Cognitive Interaction Technology Excellence Center during DA's doctoral studies (277, German Research Foundation, DFG).

## Conflict of Interest

The authors declare that the research was conducted in the absence of any commercial or financial relationships that could be construed as a potential conflict of interest.

## Publisher's Note

All claims expressed in this article are solely those of the authors and do not necessarily represent those of their affiliated organizations, or those of the publisher, the editors and the reviewers. Any product that may be evaluated in this article, or claim that may be made by its manufacturer, is not guaranteed or endorsed by the publisher.

## Footnotes

^1^Previous studies have varied stimulus onset asynchrony in sentence reading (Holderbaum and de Salles, 2011; Dambacher et al., 2012), lexical decision (de Groot et al., 1986; Perea and Gotor, 1997; Perea and Rosa, 2000), and semantic priming (Anderson and Holcomb, 1995; Holderbaum and de Salles, 2011). The outcome of these studies showed that various temporal offsets of visual and linguistic cues can modulate language processing.

^2^In another reading eye-tracking study, Sturt (2003) showed that participants read sentences with gender incongruence at the local subject slower than sentences with gender-congruent local subjects.

^3^The side of the objects in the video were counterbalanced across items.

^4^The side of the objects in the video were counterbalanced across items.
